# Mutations in *PIK3CA *are infrequent in neuroblastoma

**DOI:** 10.1186/1471-2407-6-177

**Published:** 2006-07-05

**Authors:** Vincent Dam, Brian T Morgan, Pavel Mazanek, Michael D Hogarty

**Affiliations:** 1 Division of Oncology, The Children's Hospital of Philadelphia; Philadelphia, PA, USA; 2 Department of Pediatrics, The University of Pennsylvania School of Medicine, Philadelphia, PA, USA; 3 Pediatric Oncology Department, University Children's Hospital Brno, Brno, Czech Republic

## Abstract

**Background:**

Neuroblastoma is a frequently lethal pediatric cancer in which *MYCN *genomic amplification is highly correlated with aggressive disease. Deregulated *MYC *genes require co-operative lesions to foster tumourigenesis and both direct and indirect evidence support activated *Ras *signaling for this purpose in many cancers. Yet *Ras *genes and *Braf*, while often activated in cancer cells, are infrequent targets for activation in neuroblastoma. Recently, the *Ras *effector *PIK3CA *was shown to be activated in diverse human cancers. We therefore assessed *PIK3CA *for mutation in human neuroblastomas, as well as in neuroblastomas arising in transgenic mice with *MYCN *overexpressed in neural-crest tissues. In this murine model we additionally surveyed for *Ras *family and *Braf *mutations as these have not been previously reported.

**Methods:**

Sixty-nine human neuroblastomas (42 primary tumors and 27 cell lines) were sequenced for *PIK3CA *activating mutations within the C2, helical and kinase domain "hot spots" where 80% of mutations cluster. Constitutional DNA was sequenced in cases with confirmed alterations to assess for germline or somatic acquisition. Additionally, *Ras *family members (*Hras1*, *Kras2 *and *Nras*) and the downstream effectors *Pik3ca *and *Braf*, were sequenced from twenty-five neuroblastomas arising in neuroblastoma-prone transgenic mice.

**Results:**

We identified mutations in the *PIK3CA *gene in 2 of 69 human neuroblastomas (2.9%). Neither mutation (R524M and E982D) has been studied to date for effects on lipid kinase activity. Though both occurred in tumors with *MYCN *amplification the overall rate of *PIK3CA *mutations in *MYCN *amplified and single-copy tumors did not differ appreciably (2 of 31 versus 0 of 38, respectively). Further, no activating mutations were identified in a survey of *Ras *signal transduction genes (including *Hras1*, *Kras2*, *Nras*, *Pik3ca*, or *Braf *genes) in twenty-five neuroblastic tumors arising in the *MYCN-*initiated transgenic mouse model.

**Conclusion:**

These data suggest that activating mutations in the Ras/Raf-MAPK/PI3K signaling cascades occur infrequently in neuroblastoma. Further, despite compelling evidence for *MYC *and *RAS *cooperation in vitro and in vivo to promote tumourigenesis, activation of *RAS *signal transduction does not constitute a preferred secondary pathway in neuroblastomas with *MYCN *deregulation in either human tumors or murine models.

## Background

Neuroblastoma is a common childhood tumor arising from neural crest-derived cells of the sympathetic nervous system. It is one of the most extensively characterized solid tumors at the genomic level and provides the paradigm for the clinical utility of genomic alterations in defining tumor phenotype. Much of the clinical heterogeneity seen with this tumor (spanning spontaneous regression to relentless disease progression) can be attributed to distinct tumor-specific genetic alterations that correlate strongly with disease course. These include genomic loss within 1 p and 11 q, and gain of 17 q, among others. Still, little is known about the genes or cellular processes targeted by the majority of these genomic changes. Key regulatory genes frequently targeted for activation or inactivation in a plethora of adult human cancers (e.g., *Ras*, *TP53*, *CDKN2A*, *RB1*) are rarely, if ever, mutated in neuroblastoma. The sole exception to this is the *MYCN *proto-oncogene, which is markedly amplified and overexpressed in ~22% of neuroblastomas and independently correlates with advanced disease and adverse outcome [1,2].

Deregulated expression of *MYC *genes, including *MYCN*, inhibits cellular differentiation and promotes mitogen-independent proliferation [3]. However, sensitivity to apoptotic stressors is concurrently enhanced as a safeguard against neoplastic transformation, and therefore oncogenesis mediated by *MYC *genes requires co-operating lesions that either disable the *MYC*-death signaling axis [4,5] or repress it through constitutive survival signals. We are interested in defining these co-operating lesions, in addition to identifying oncogenic lesions that contribute to neuroblastoma tumourigenesis or progression independent of *MYCN*. Activating mutations in *Ras *family genes have been identified in 30% of human cancers [6]. Activated *Ras *(1) co-operates with *MYC *to transform primary cells in vitro and promote tumourigenesis in vivo [7]; (2) regulates myriad cellular processes including survival; and (3) acts to stabilize Myc proteins through post-translational modifications [8,9]. These data strongly support this pivotal pathway as a candidate for aberrant activation in human neuroblastoma, and specifically in co-operating with *MYCN *in tumors with *MYCN *amplification or deregulated expression.

Still, activating mutations have rarely been identified in *HRAS1*, *KRAS2 *or *NRAS *in human neuroblastoma [10-12]. Occasional Ras effector alterations have been identified but collectively they are infrequent. These include biallelic inactivation of *NF1 *and mutations in the Noonan-associated *SHP2/PTPN11 *gene [13-16]. Downstream of Ras are multiple signal transduction pathways responsible for the execution of Ras-mediated cellular effects [17] and cell-type and species specificity for transformation has been noted [18]. The most widely validated as contributing to human tumorigenesis include the Raf-MAPK and phosphatidylinositol 3-kinase (PI3K) pathways [19,20]. Somatic activating mutations have been identified in multiple human cancers within these pathways, particularly in the *BRAF *and *PIK3CA *genes, respectively [21,22]. Activated PI3K signaling has been proposed to foster Myc/Ras co-operativity through Akt-mediated phosphorylation of FoxO transcription factors [23] to de-represses *MYC *target genes, and activated PI3K genes protect against Myc induced cell death sensitivity [24]. These Ras effector signaling components have not been studied in neuroblastoma. As *PIK3CA *has been shown to be activated through somatic mutation in >25% of colorectal, breast and ovarian carcinomas, as well as in human CNS tumors (including anaplastic oligodendrogliomas, glioblastoma multiforme and medulloblastoma), we hypothesized that *PIK3CA *mutations may substitute for activating *RAS *lesions in neuroblastoma tumourigenesis. Therefore, we performed a targeted sequencing analysis of the *PIK3CA *gene in primary neuroblastomas and neuroblastoma-derived cell lines.

To further explore Raf-MAPK and PI3K signaling in neuroblastic tumors we evaluated tumors arising in neuroblastoma-prone genetically engineered mice (GEM). In this model human *MYCN *gene overexpression is targeted to neural crest tissues using the rat tyrosine hydroxylase promoter [25]. Neuroblastic tumors arise stochastically within the sympathetic ganglia with near-complete penetrance. These tumors are genomically complex with chromosomal gains and losses at regions orthologous to regions similarly altered in human neuroblastoma [26,27]. Thus, the model faithfully recapitulates the human condition with deregulated *MYCN *as the initiating event biologically and genetically [25-27]. To determine whether alterations in Ras/Raf/PIK signaling were co-operating with *MYCN *in this model we sequenced *Hras1*, *Kras2*, *Nras*, *Braf *and *Pik3ca *to assess for activating mutations as these genes and pathways have not been studied previously using this model.

## Methods

### Human neuroblastoma specimens

Forty-two (42) primary neuroblastoma-derived DNA specimens were acquired from the Children's Oncology Group (COG; Arcadia, CA) for *PIK3CA *sequence analysis. Constitutional DNA samples were subsequently obtained from the COG Nucleic Acids bank for two patients with tumors demonstrating *PIK3CA *sequence alterations to determine whether these were tumor-specific (somatic) or germ line in origin. These studies were approved by the Institutional Review Board at The Children's Hospital of Philadelphia. Twenty-seven (27) additional neuroblastoma cell line DNA specimens, obtained from The Children's Hospital of Philadelphia cell line bank and the Wellcome Trust Sanger Institute and reported previously [28,29], were similarly investigated for *PIK3CA *mutations.

### Murine neuroblastoma specimens

Twenty-five (25) tumors were harvested from genetically engineered mice overexpressing human *MYCN *in neural-crest tissues under the rat tyrosine-hydroxylase promoter [*TH-MYCN *GEM model] [25]. In this model, tumors arise stochastically within the peripheral sympathetic nervous system (in the retroperitoneum or posterior thorax) with near-complete penetrance in mice homozygous for the *MYCN *transgene. Founders for establishment of the *TH-MYCN *GEM colony at The Children's Hospital of Philadelphia were generously provided by Dr. William Weiss (UCSF). Ten tumors in this study came from early backcrosses (received from UCSF) and fifteen additional tumors came from animals backcrossed to homozygosity in the 129X1/SvJ background. Tumors were flash frozen at necropsy and partitioned for subsequent DNA and RNA extraction using the DNeasy and RNeasy Midi Tissue kits, respectively, according to the manufacturer's instructions (Qiagen; Valencia, CA). All mouse experiments were approved by the Institutional Animal Care and Use Committee (IACUC) at The Children's Hospital of Philadelphia.

### Candidate gene sequencing: *human PIK3CA*

Primer pairs that amplify exons 4, 5, 9 and 20 of the *PIK3CA *gene in human tumors were synthesized (Integrated DNA Technologies, Inc., Coralville, 1A). These exons encode the C2, helical, and kinase domains, respectively, which demonstrate high rates of somatic mutation in human cancers [22]. The four primer pairs used to amplify exons 5, 9 and 20 from genomic DNA were identical to those used by Samuels, et al. [22]. Primers that amplify exon 4 were designed using Primer 3 software : HumPIK3CA-ex4-F1, 5'-TCT TGT GCT TCA ACG TAA ATC C-3', and HumPIK3CA-ex4-R1, 5'-CGG AGA TTT GGA TGT TCT CC-3'. PCR amplification with each pair yielded a single specific band of appropriate size using control genomic DNA input.

### Murine Pik3ca

Primer pairs that amplify exons 9 and 20 (helical and kinase domains, respectively) were designed using Primer 3 software and *Pik3ca *sequence (accession no. GenBank:XM355754). These were: (1) MurPIK3CA-ex9-F1, 5'-CCA AGG AAA TCA TGG CAG AG-3', and MurPIK3CA-ex9-R1, 5'-TGT CCC TGA CAG GAA GAA GG-3'; and (2) MurPIK3CA-ex20-F1, 5'-GCA GCT CAC TGA CCA GAT GT-3', and MurPIK3CA-ex20-R1, 5'-ACT GCC ATG CAG TGG AGA A-3'. Amplification of genomic DNA (control) yielded appropriate single products of 240 and 358 base pairs, respectively.

### Murine Ras/Raf pathway genes

Primer sequences to amplify the entire coding region of murine *Hras1 *(GenBank:NM008284), *Kras2 *(GenBank:NM021284), and *Nras *(GenBank:NM010937) from cDNA were kindly provided by Lewis Chodosh (University of Pennsylvania). These are: (1) mHRAS1-F1-seq, 5'-AGA ATA CAA GCT TGT GGT GGT GG-3', and mHRAS1-R1-seq, 5'-CCT GTA CTG ATG GAT GTC CTC G-3'; (2) mKRAS2-F1-seq, 5'-GTA AGG CCT GCT GAA AAT G-3', and mKRAS2-R1-seq, 5'-GGT GAA TAT CTT CAA ATG AT-3'; and (3) mNRAS-F1-seq, 5'-GGT CTC CAA CAG CTC AGG TTG AAG-3', and mNRAS-R1-seq, 5'-GTA CCT GTA GAG GTT AAT ATC TGC A-3'. Primers to amplify the serine/threonine kinase domain of *Braf *(XM355754) were designed using Primer 3 software, and were: mBRAF-F2, 5'-GAA TGT GAC AGC ACC CAC AC-3', and mBRAF-R1, 5'-CGG GTT TTT ATC TTG CAT TC-3'. All primer pairs amplified a single reaction product of predicted size from control cDNA.

### PCR amplification, sequencing and mutation detection

Two PCR reactions were carried out for each sequencing reaction in a final volume of 20 μl. The reaction mixture consisted of 5 μl stock Master Mix (16 ml TaqGold Buffer II, 9.6 ml 25 mM MgCl_2_, 5.2 ml 2% Ficoll, 200 μl 0.025% Bromophenol Blue, 1.6 ml 10 nM dNTP Mix, and 7.4 ml ddH_2_O), 0.4 μl 20 μM Forward-primer, 0.4 μl 20 μM Reverse-primer, 13.0 μl sterile PCR-grade water, 0.2 μl TaqGold DNA Polymerase (Promega Corporation, Madison, Wl), and 1.0 μl sample template. For sequencing from DNA, 80 ng were used as template input. For sequencing from cDNA, 2 μg RNA was reverse-transcribed in a final volume of 20 μl with the oligodeoxythymidylic acid primer using the Superscript II pre-amplification system according to the manufacturer's instructions (Life Technologies, Gaithersburg, MD). One μl of reverse transcribed cDNA was used as a template. The following PCR protocol was utilized for each sample and carried out in a Programmable Thermal Controller-100 (MJ Research, Inc., Waltham, MA): (1) 94°C × 12 minutes (polymerase activation), (2) thirty DNA polymerization cycles of 94°C × 45 seconds, 55°C × 45 seconds, 72°C × 45 seconds, followed by 72°C × 10 minutes and 4°C holding temperature. The individual PCR amplifications for each reaction were combined and electrophoresed through a 1% agarose gel in 1× TBE buffer for 40 minutes at 90 V. The PCR product was verified as a single specific band of predicted size then extracted and purified using the QIAQuick Gel Extraction Kit (Qiagen Inc., Valencia, CA).

All amplification products were sequenced bi-directionally in an automatic sequencer (Applied Biosystems, Foster, CA) using the primary forward and reverse primers. This provided at least double coverage of all evaluated sequence in each tumor sample. Sequence analysis was carried out using Sequencher version 4.1.4 (Gene Codes Corporation, Ann Arbor, MI) in addition to a manual chromatogram review. Confirmatory resequencing from replicate PCR amplification reactions was performed for any sequence that was ambiguous or deviated from wild-type so that all abnormal sequences were confirmed in at least quadruplicate from replicate amplification reactions.

## Results and discussion

### *PIK3CA *mutation screening in human neuroblastoma

**Figure 1:**
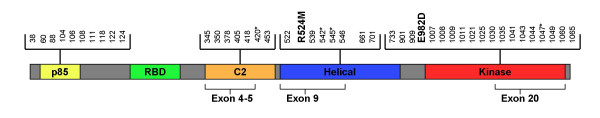
***PIK3CA *structure**. Schematic diagram of Pik3ca protein illustrating functional domains and mutations (grouped by domain) reported to date [22, 30, 32, 33, 40, 41]. Codons with frequent missense mutations reported ('hotspots') are denoted by asterisks. Location of experimentally found mutations in this study are denoted in boldface. p85, PI3K p85 regulatory subunit binding domain; RBD, Ras binding domain; C2, calcium-dependent phospholipid-binding domain; Helical, PI3K helical domain; Kinase, PI3/4-kinase domain.

We screened 69 human neuroblastomas (42 primary tumors and 27 neuroblastoma-derived cell lines) for *PIK3CA *gene mutations within exons 4, 5, 9 and 20 (Tables 1 and 2). *PIK3CA *is one of the most commonly mutated oncogenes in human cancers, occurring at high prevalence in tumors of the colon, breast, ovary, stomach and liver, and at lower prevalence in multiple other cancer types. Exons 9 and 20 encode portions of the helical and kinase domains, respectively, that ~80% of previously reported *PIK3CA *mutations cluster within [22,30]. These regions encode critical domains mediating lipid kinase activity [31]. Exons 4 and 5 encode the C2 region of the protein (calcium-dependent phospholipid binding domain) that is mutated in an additional ~5% of tumors, yet this region accounts for 50% of mutations reported in glioblastoma multiforme, a highly malignant neural tumor [22], so we included it in our neuroblastoma investigations (Figure 1).

**Figure 2:**
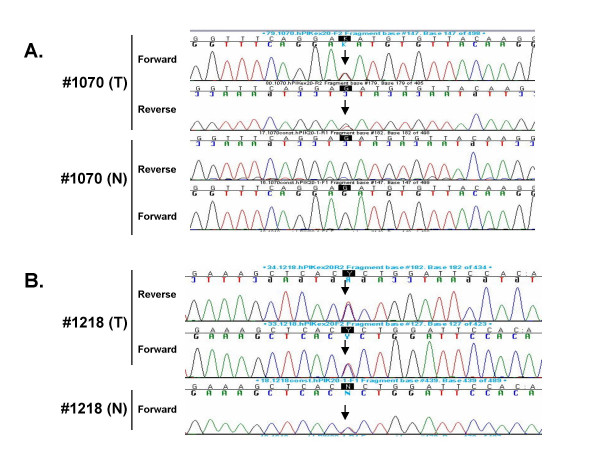
**Detection of *PIK3CA *mutations**. Chromatogram tracing for exon 20 sequences encoding the catalytic kinase domain in primary neuroblastomas #1070 (A) and #1218 (B). Sequences demonstrate heterozygous alterations in these tumor samples as depicted by the arrows, with the amino acid change for #1070 being GAG (glutamic acid) to GAT (representing GAU, aspartic acid). Alterations were confirmed bi-directionally and in replicate sequencing. T, tumor DNA sequence; N, normal (or constitutional) DNA sequence. Note the tumor-specific (somatic) mutation in #1070, whereas the alteration in the 3'-UTR in #1218 is present in both tumor and constitutional DNA and likely represents a rare polymorphic variant.

Overall, *PIK3CA *mutations were infrequently found, with non-synonymous coding sequence alterations being identified in 2 of 69 specimens (2.9%). An additional 16 tumors had sequence alterations identified in non-coding regions (including three specific alterations in introns and one in the 3'-untranslated region). Of the coding sequence mutations, one was identified within the helical domain encoded within exon 9, occurring in the *MYCN *amplified neuroblastoma cell line NLF. This heterozygous missense mutation (G to T) results in an arginine to methionine substitution (R524M). This mutation has not to our knowledge been reported previously and is of undetermined functional significance. No constitutional nor primary tumor DNA was available from the patient the cell line was derived from so it could not be directly confirmed that this was a somatic mutation, nor whether the mutation occurred in vivo or ex vivo during the course of tissue culture propagation. The second mutation was identified within the kinase domain (Figure 2A). This was a heterozygous G to T alteration leading to a glutamic acid to aspartic acid substitution (E982D) in primary tumor #1070 (INSS stage 3, *MYCN *amplified). Constitutional DNA from this individual was wild-type, confirming the kinase domain mutation to be tumor-specific (somatic). This specific mutation has not been previously described nor functionally characterized to date, although an E982K mutation has been found in anaplastic thryroid carcinoma [32].

In addition, four unique sequence alterations were identified in at least one specimen each in non-coding regions: two within intron 8 (C19339T and G19361A [nucleotide number refers to position in genomic sequence relative to the first nucleotide of exon 1]), one within intron 9 (T19623G), and one within the 3'-UTR (heterozygous T35577C). These were identified in 5 (7.2%), 1 (1.4%), 9 (13%) and 1 (1.4%) sample, respectively. The 3'-UTR alteration was identified in primary tumor #1218. Constitutional DNA from this patient was sequenced and demonstrated the same alteration, suggesting this represents a polymorphic variant (Figure 2B). None of these alterations appears to disrupt splice donor or acceptor sites. However, none of these alterations represents a previously identified SNP based on a query of the dbSNP (build 125; ). Finally, no mutations were identified within exons 4 or 5 within the C2 domain in any neuroblastoma tissue.

Thus, *PIK3CA*, like *RAS *genes, is an infrequent target for activating mutation in neuroblastoma. In our series of 69 human neuroblastomas, amino acid sequence alterations were identified in only two (one in a *MYCN *amplified cell line, and one in a *MYCN *amplified INSS stage 3 tumor), albeit in a survey restricted to domain "hotspots" (C2, helical, kinase) that harbor >80% of mutations identified to date [22]. Although these alterations occur within regions where mutations cluster, it is not known whether they augment lipid kinase activity and are functionally significant. Still, we did not identify any synonymous sequence alterations in the coding regions assessed so random non-selected alterations are infrequent. Of note, these findings are consistent with similar low frequencies of mutation (<5%) in other embryonal malignancies such as medulloblastoma [33] and leukemia/lymphomas [30], in contrast to the higher prevalence of mutations in many adult epithelial cancers. The prevalence of *PIK3CA *mutations in other pediatric cancers has not yet been reported.

### Assessment of the *Ras*, *Raf*, *PI3K *pathway in murine neuroblastomas arising in the TH-MYCN genetically engineered mouse (GEM)

Neuroblastomas arise with near-complete penetrance in TH-MYCN GEM homozygous for the transgene. Tumors develop focally in the peripheral nervous system and demonstrate genomic complexity with high similarity to human neuroblastomas [26]. For this reason, we used this model to determine whether *Ras*, *Raf *and/or *Pi3k *signaling constituted a preferred secondary pathway with *MYCN *deregulation. To assess for activating mutations in *Pi3kca *we sequenced the helical and kinase encoding regions (exons 9 and 20) from 25 neuroblastomas harvested from heterozygous or homozygous transgene carrying mice. All 25 murine neuroblastomas harbored wild-type *Pik3ca *sequence (Table 3).

We next assessed genes in the *Ras *and *Raf *pathway in these same 25 murine neuroblastomas, including *Hras1*, *Kras2*, *Nras *and *Braf*, which are frequently mutated in human cancers and in *MYC*-initiated murine tumor models [34]. No non-synonymous sequence alterations were identified in these genes. One tumor (THMYCN-21) had three synonymous alterations identified: one each in the *Hras1*, *Kras2 *and *Nras *genes. One additional synonymous alterations was identified in THMYCN-17 in *Kras2 *(Table 3). These findings likely represent polymorphic variants and were found only in tumors from early mice backcrosses predicted to harbor strain specific polymorphisms. Tumors harvested following extensive backcrossing to homozygosity had no non-synonymous alterations identified. The low frequency of *Pik3ca*, *Ras *family, and *Braf *activating mutations in tumors arising in the TH-MYCN GEM supports that activated Ras-mediated signal transduction is not a pre-requisite for neuroblastoma development in concert with deregulated *MYCN *in this model. In this respect the GEM model is consistent with the human tumor.

## Conclusion

*Pik3ca*, *Ras *family, and *Braf *activating mutations occur rarely in human neuroblastomas or in a murine model driven by neural-restricted *MYCN *overexpression. These data support that activated Ras-mediated signal transduction does not constitute a preferred secondary pathway for neuroblastic tumors in concert with deregulated *MYCN*. This despite the potent cooperation between *Myc *and activated *Ras *genes demonstrable in vitro and in vivo to induce transformation and/or tumorigenicity. Further, these tumor promoting pathways are rarely targeted independent of *MYCN *deregulation as demonstrated by the absence of activating lesions in neuroblastoma tumors and cell lines without *MYCN *amplification. Tumor induction through activation of Ras/Raf-MAPK/PI3K pathways clearly demonstrates cell-type specificity. For example, studies have demonstrated epithelial cell proliferation being augmented by *Ras *activation while mesenchymal cells (e.g., fibroblasts) demonstrate growth inhibition [35]. This specificity may underscore the variable frequency of Ras pathway mutation selection across cancers and partly explain the paucity of activating mutations found in non-epithelial embryonal malignancies such as neuroblastoma.

However, it remains strictly possible that activating mutations in alternative *Ras *signaling constituents occur in neuroblastoma. Less studied family members such as *MRAS *[36] and *RRAS *[37] have oncogenic potential in mammalian cells and the former demonstrates restricted expression to neural tissues. These have not been investigated in neuroblastoma although both are expressed in primary neuroblastomas at the mRNA level (data not shown). In distinction to activating point mutations, pathway activation through *PIK3CA *gene dosage effects following low level genomic amplification have also been described [38]. However, these appear predominantly in those epithelial carcinomas (breast, ovarian, gastric carcinoma) in which activating mutations occur at higher frequency as well. In contrast, gain of 3q26 including the *PIK3CA *locus is infrequently reported in neuroblastoma cells, including in those cell lines studied herein [28]. Finally, additional effector arms to Ras-mediated signaling may play a role in tumourigenesis. These include Tiam-1, RalGDS, RASSF and others (reviewed in [17]). *RASSF1A *has been shown to be epigenetically silenced in a significant proportion of high-risk neuroblastomas [39] and elimination of this potentially pro-apoptotic pathway may be more critical than activation of proliferative or survival signaling in the establishment of embryonal cancers. Alternatively, *Ras *independent secondary pathways may be preferentially co-opted in neuroblastoma tumor initiation. These pathways remain elusive but certainly warrant further investigation, as defining the preferred pathways deregulated in neuroblastoma might provide suitable novel targets for directed therapies against this frequently lethal tumor.

## Abbreviations

INSS: International Neuroblastoma Staging System; MAPK: mitogen-activated protein kinase.

## Competing interests

The author(s) declare that they have no competing interests.

## Authors' contributions

VD and BM carried out the molecular analysis and performed data analyses of *PIK3CA *in both human and murine tumors. PM carried out the molecular analysis and performed data analyses for *Ras *family and *Raf *genes in the murine model. MDH conceived of the study, directed its design, coordination and data analysis and edited the manuscript. All authors read and approved the final manuscript.

## Pre-publication history

The pre-publication history for this paper can be accessed here:



## Figures and Tables

**Table 1:** *PIK3CA *in Primary Human Neuroblastomas

**Primary NB**	**Age (mos.)**	**INSS stage**	***MYCN***	Tumor *PIK3CA *status*	**Constitutional *PIK3CA *status**
#1347	1.4	1	S	wt (het. G19361A; intron 8)	-
#1354	10.5	1	S	wt	-
#1344	11.7	1	S	wt	-
#1260	13.5	1	S	wt	-
#1308	23.2	1	S	wt	-
#1334	25.5	1	S	wt (het. T19623G; intron 9)	-
#1127	52.8	1	S	wt	-
#354	71.9	1	AMP	wt (het. C19339T; intron 8)	-
#1248	2.3	2	S	wt	-
#1087	16.4	2	S	wt	-
#1142	48.7	2	S	wt	-
#1281	82.9	2	S	wt	-
#190	104.0	2	AMP	wt	-
#1330	20.0	2	S	wt	-
#1339	50.0	2	S	wt	-
#0157	178.1	2	AMP	wt (het. C19339T; intron 8)	-
#1137	4.5	3	AMP	wt	-
#1290	7.9	3	S	wt	-
#1345	12.2	3	S	wt	-
#1258	21.1	3	S	wt	-
#1070	23.4	3	AMP	**E982D **(+het. C19339T; intron 8)	wt (+het. C19339T; intron 8)
#1223	35.8	3	AMP	wt	-
#1289	2.5	4	S	wt	-
#1302	2.6	4	S	wt (het. T19623G; intron 9)	-
#1259	3.2	4	S	wt (het. C19339T; intron 8)	-
#1066	6.7	4	AMP	wt (het. C19339T; intron 8)	-
#1304	8.9	4	S	wt	-
#1068	13.5	4	AMP	wt	-
#1243	19.2	4	AMP	wt	-
#1266	23.3	4	AMP	wt	-
#1198	24.0	4	AMP	wt	-
#1287	24.9	4	AMP	wt (het. T19623G; intron 9)	-
#1210	33.4	4	S	wt	-
#1204	36.8	4	S	wt	-
#1218	37.8	4	S	wt (het. T35577C in 3'-UTR)	wt (het. T35577C in 3'-UTR)
#1213	40.5	4	S	wt	-
#1263	40.7	4	AMP	wt	-
#1236	46.3	4	S	wt	-
#1337	50.0	4	S	wt	-
#1246	52.1	4	AMP	wt	-
#1288	54.2	4	AMP	wt (het. T19623G; intron 9)	-
#1220	60.3	4	S	wt	-

**INSS**; International Neuroblastoma Staging System.

**AMP**; *MYCN *genomic amplification.

**S**; single-copy *MYCN *per haploid genome.

**wt**; wild-type sequence

* Nucleotide position referenced from genomic sequence 1st nucleotide of exon 1.

**Table 2:** *PIK3CA *in Neuroblastoma cell lines

**NB Cell Line**	***MYCN***	**Exon 4**	**Exon 5**	**Exon 9**	**Exon 20**
LA-N-1	AMP	wt	wt	wt	wt
SK-N-BE2	AMP	wt	wt	wt	wt
SK-N-SH	S	wt	wt	wt	wt
NB-69	S	wt	wt	wt	wt
SK-N-DZ	AMP	wt	wt	wt	wt
SMS-KCN	AMP	wt	wt	wt	wt
SY5Y	S	wt	wt	wt (T19623G; intron 9)	wt
CHP134	AMP	wt	wt	wt	wt
CHP126	AMP	wt	wt	wt	wt
CHLA-150	S	wt	wt	wt	wt
NGP	AMP	wt	wt	wt	wt
SMS-KAN	AMP	wt	wt	wt (T19623G; intron 9)	wt
IMR5	AMP	wt	wt	wt	wt
NMB	AMP	wt	wt	wt (T19623G; intron 9)	wt
SK-N-AS	S	wt	wt	wt	wt
LA-N-6 (OAN)	S	wt	wt	wt	wt
CHLA-79	S	wt	wt	wt (T19623G; intron 9)	wt
IMR32	AMP	wt	wt	wt	wt
NB16	S	wt	wt	wt	wt
SK-N-FI	S	wt	wt	wt (T19623G; intron 9)	wt
NBL-S	S	wt	wt	wt	wt
CHP901	AMP	wt	wt	wt	wt
CHLA-171	S	wt	wt	wt	wt
SMS-KCNR	AMP	wt	wt	wt	wt
SMS-SAN	AMP	wt	wt	wt	wt
CHLA-123	AMP	wt	wt	wt	wt
NLF	AMP	wt	wt	**G>T/G; Het. R524M**	wt

**wt**; wild-type sequence.

**AMP**; *MYCN *amplified.

**S**; *MYCN *single-copy per haploid genome.

* Position referenced from genomic sequence 1st nucleotide of exon 1.

**Table 3:** *Ras family*, *Braf *and *PIK3CA *in Murine Neuroblastomas

TH-MYCN NB*	MYCN**	***Hras1***	***Kras2***	***Nras***	***Braf***	***PIK3CA***
1	+++	wt	wt	wt	wt	wt
2	+++	wt	wt	wt	wt	wt
3	+++	wt	wt	wt	wt	wt
4	+++	wt	wt	wt	wt	wt
5	+++	wt	wt	wt	wt	wt
6	+++	wt	wt	wt	wt	wt
7	+++	wt	wt	wt	wt	wt
8	+++	wt	wt	wt	wt	wt
9	+++	wt	wt	wt	wt	wt
10	+++	wt	wt	wt	wt	wt
11	+++	wt	wt	wt	wt	wt
12	+++	wt	wt	wt	wt	wt
13	+++	wt	wt	wt	wt	wt
14	+++	wt	wt	wt	wt	wt
15	+++	wt	wt	wt	wt	wt
16	+++	wt	wt	wt	wt	wt
17	+++	wt	**Y32Y**	wt	wt	wt
18	+++	wt	wt	wt	wt	wt
19	+++	wt	wt	wt	wt	wt
20	+++	wt	wt	wt	wt	wt
21	+++	**S65S**	**Y32Y**	**A18A**	wt	wt
22T	+++	wt	wt	wt	wt	wt
23T	+++	wt	wt	wt	wt	wt
24T	+++	wt	wt	wt	wt	wt
25	+++	wt	wt	wt	wt	wt

**wt**; wild-type sequence.

* T denotes thoracic tumor (all others are retroperitoneal/abdominal).

** MYCN transgene overexpressed in neural crest tissues.
